# Weathering a Cytokine Storm: A Case of EBV-Induced Hemophagocytic Lymphohistiocytosis

**DOI:** 10.1177/2324709616647409

**Published:** 2016-05-02

**Authors:** Tiffany Y. Shaw, Michael Schivo

**Affiliations:** 1 University of California, Davis, Sacramento, CA, USA

**Keywords:** HLH, multiorgan dysfunction syndrome, sepsis, macrophage activation syndrome, hyperferritinemia

## Abstract

Hemophagocytic lymphohistiocytosis (HLH) is a rare but life-threatening disease caused by excessive immune activation. Acquired HLH is seen in adults and is often caused by infection or malignancy. Diagnosis is difficult and usually missed as clinical and laboratory findings are nonspecific. Moreover, the pathophysiology of the systemic inflammatory response syndrome and/or sepsis is remarkably similar to HLH. Thus, in patients presenting with presumed severe sepsis or septic shock complicated by multiorgan failure without a clear infectious source, HLH should be considered. A disproportionately high ferritin may be one of the earlier laboratory findings to suggest HLH. We discuss a case of a young male who presented with presumed septic shock with multiorgan failure who was eventually found to have Epstein-Barr virus–induced HLH.

## Introduction

Hemophagocytic lymphohistiocytosis (HLH) is a rare but life-threatening disease caused by uncontrolled immune activation leading to excessive macrophage activity and cytokine release.^1^ Tissue infiltration by overactive macrophages and cytotoxic lymphocytes leads to multiorgan dysfunction.^1^ There are 2 types of HLH: primary or familial HLH, most commonly seen in infants and young children and caused by genetic defects,^2^ and secondary or acquired HLH, seen in adults and often triggered by infection, malignancy, or rheumatologic disease.^1,2^

Secondary HLH is difficult to identify, and a diagnosis is often missed as it can mimic the systemic inflammatory response syndrome or sepsis. Furthermore, HLH has a variable presentation with nonspecific clinical symptoms and laboratory findings.^2^ Because of probable underdiagnosis, the true incidence and prevalence of HLH in adults are unknown given insufficient epidemiologic data.^2,3^ If left untreated the disease is frequently fatal, with median survival estimated to be less than 2 months.^4^ With this case presentation, our aim is to increase awareness and help facilitate early recognition of HLH.

## Case Report

A 29-year-old Hmong male with no significant medical history presented to the emergency department with a 6-month history of progressive abdominal pain, substantial weight loss, and weakness. He declined medical attention initially; however, his symptoms worsened and he re-presented with progressive confusion. Initially, the patient was alert but severely cachectic, jaundiced, and hypotensive with a blood pressure of 72/46 mm Hg. Physical examination revealed diffuse skin petechiae and splenomegaly.

Laboratory studies indicated multiorgan failure, including liver, kidney, and bone marrow failure with severe pancytopenia (Table 1). His chest X-ray showed bilateral infiltrates concerning for the acute respiratory distress syndrome, and his oxygen saturation fell to 54% prompting intubation and mechanical ventilation for hypoxemic respiratory failure. Multiple vasopressors and broad-spectrum antibiotics were initiated for presumed septic shock secondary to pneumonia, and he was placed on emergent continuous hemodialysis for a refractory metabolic acidosis. Initial evaluation for underlying HIV, tuberculosis, and acute infectious hepatitis were negative. A polymerase chain reaction (PCR)–based respiratory viral panel was unremarkable, body fluid cultures were negative, and no definite infectious source was identified. On hospital day 4, a bone marrow biopsy was performed given his profound pancytopenia, and the initial results showed no overt malignant infiltrates.

**Table 1.** Trend of Laboratory Studies During Hospital Admission.

		Hospital Day	
	1	12	18
AST (U/L)	504	65	56
ALT (U/L)	247	307	51
ALP (U/L)	826	307	341
Total bilirubin (mg/dL)	13.9	32.4	34.2
Albumin (g/dL)	<1.0	2.7	2.9
INR	1.89	1.33	
BUN (mg/dL)	99	59	44
Creatinine (mg/dL)	1.67	1.19	0.50
WBC (K/mm^3^)	1.5	1.2	1.9
Hgb (g/dL)	3.8	8.2	7.4
Platelets (K/mm^3^)	6	8	7
Ferritin (ng/mL)	13 444	2909	3118

Abbreviations: AST, aspartate aminotransferase; ALT, alanine aminotransferase; ALP, alkaline phosphatase; INR, international normalized ratio; BUN, blood urea nitrogen; WBC, white blood cell count; Hgb, hemoglobin.

**Figure 1.**
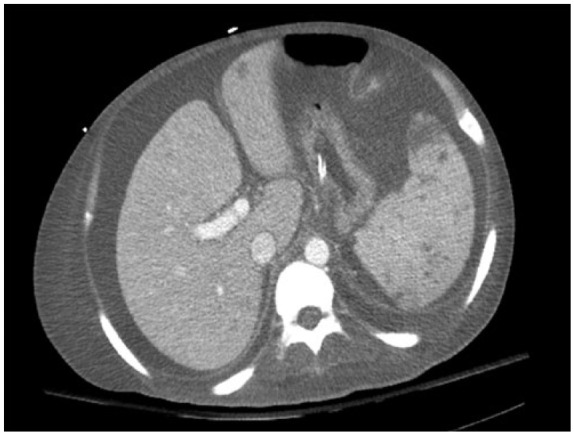
Abdominal computed tomography scan. Here splenomegaly, multiple hepatic and splenic lesions, and lymphadenopathy are seen.

**Figure 2.**
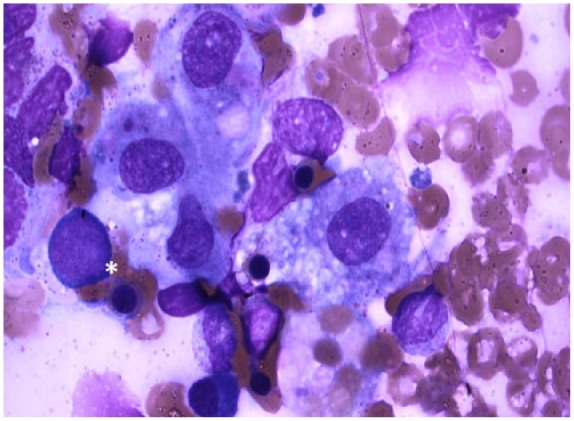
Bone marrow biopsy. Giemsa stain showing hemophagocytosis (*).

**Figure 3.**
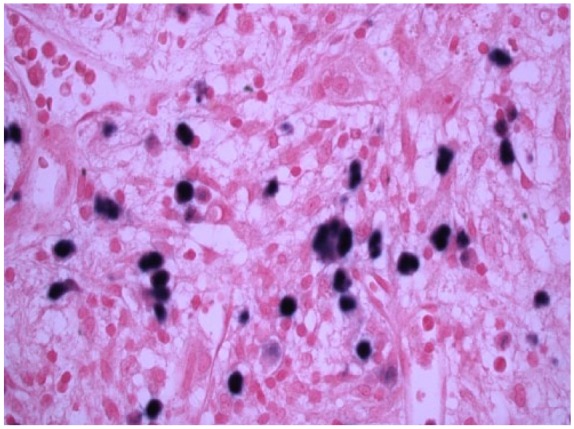
Epstein-Barr virus (EBV)–encoded RNA-1 in situ hybridization of the bone marrow core biopsy. The dark stains are EBV-positive cells.

An abdominal computed tomography scan demonstrated splenomegaly, multiple liver and splenic lesions, and lymph node adenopathy (Figure 1). Family declined biopsy due to the patient’s clinical deterioration and thus malignancy was not excluded. Continued evaluation of his pancytopenia revealed a ferritin level of 13 444 ng/mL, raising the concern for HLH. Additional laboratory data demonstrated low fibrinogen levels at 104 mg/dL (normal range = 179-395 mg/dL) and high soluble IL-2 receptor levels of 10 210 pg/mL (normal range ≤1033 pg/mL). Rheumatologic workup included ANA, anti–mitochondrial antibody, and anti–smooth muscle antibody, all of which were negative. The infectious workup was negative for cytomegalovirus, herpes simplex virus, *Varicella, Pneumocystis jiroveci, Brucella*, histoplasmosis, and leptospirosis; however, his blood Epstein-Barr virus (EBV) PCR was positive. Further evaluation of his bone marrow biopsy showed a hypocellular marrow with histiocytic prominence and rare hemophagocytosis (Figure 2), thus completing the diagnostic criteria for HLH (Table 2). In addition, marrow EBER in situ hybridization showed many EBV-positive cells (Figure 3). The HLH 2004 treatment protocol recommends initial therapy with intravenous (IV) dexamethasone and etoposide, an anti-topoisomerase II agent. On hospital day 12, the patient was started on IV dexamethasone though etoposide was held given the patient’s worsening liver failure marked by a rising bilirubin (Table 1) and the family’s refusal given his deteriorating clinical state.^2^

**Table 2.** Criteria for Hemophagocytic Lymphohistiocytosis Diagnosis Based on the 2004 Trial^1^.

Five of 8 criteria must be present for diagnosis
1. Fever (>38.5°C)
2. Splenomegaly
3. Pancytopenia
a. Hemoglobin <9 g/mL
b. Platelets <100 K/mm^3^
c. Neutrophils <1 × 10^9^ L/min
4. Hypertriglyceridemia (≥265 mg/100 mL) and/or hypofibrinogenemia (≤150 mg/100 mL)
5. Ferritin ≥500 ng/mL
6. Hemophagocytosis in the bone marrow, spleen, or lymph nodes
7. Low or absent NK (natural killer) cell activity
8. Elevated soluble IL-2 receptor (>2400 U/mL) or >2 standard deviation above the age-adjusted mean

On hospital day 13, the patient was started on IV acyclovir, and the Hematology/Oncology consult service recommend therapy with rituximab given limited data suggesting benefit if used in conjunction with HLH therapy in patients specifically with EBV-induced HLH.^5^ Therefore, on hospital day 14 the patient was started on IV rituximab with a plan to start etoposide if the patient tolerated the other therapies. While the patient had a reduction in ferritin levels (Table 1), he became obtunded, remained ventilator-dependent and hypotensive despite vasopressor support, and ultimately succumbed to his disease on hospital day 19.

## Discussion

Hemophagocytic lymphohistiocytosis is a rare, life-threatening clinical syndrome that results from excessive activation of the immune system leading to uncontrolled cytokine release, tissue infiltration of histiocytes and lymphocytes, and multiorgan failure. If left untreated the median survival is estimated to be less than 2 months, with an overall mortality of 58% to 75% in adults.^1,4^ To help improve survival, early diagnosis and initiation of treatment is essential. The triggers for acquired HLH are infectious (viral, most common), malignancy (hematologic, most common), rheumatologic diseases, and unknown (idiopathic).^1-3^ It has been shown through multiple studies that malignancy-associated HLH carries a far worse prognosis than HLH associated with infections.^1-3,6,7^

Importantly, the clinical presentation of HLH is extremely variable, and often the diagnosis of HLH is missed as clinicians focus on the underlying infection or malignancy rather than the overwhelming cytokine production. As such, the true incidence and prevalence of acquired HLH is unknown.^2,3^ Interestingly, the majority of EBV-induced HLH cases have been reported in Asia. A Japanese study estimated the incidence of HLH to be 1 in 800 000 persons per year, with 90% being acquired cases and about a third of these cases were related to EBV infection.^4^ Thus, the incidence of EBV-HLH annually was estimated to be about 0.4 per million persons; however, there are insufficient data and not enough reported cases around the world to estimate incidences elsewhere.^4^ The high incidence of EBV-induced HLH in Asian countries has been hypothesized to be due to a more virulent strain of EBV; however, previous studies have not linked any specific strain of EBV to HLH.^4^ Our patient immigrated from Laos at age 9 and therefore could have initially been exposed to EBV in his home country rather than in the United States. It is not clear from the data collected if he had a particularly virulent EBV strain or if he had a uniquely robust response to EBV due to a genetic predisposition.

The exact pathophysiology of HLH is not well understood; however, EBV is one of the most common HLH triggers. A close examination of EBV-associated HLH cases may shed light on mechanisms of HLH development.^1,2,4^ One hypothesized mechanism is that EBV latent membrane protein 1 inappropriately activates cytotoxic and helper T cells leading to excessive cytokine release.^2,4^ EBV-positive T-Cell lymphoproliferative disorders have been seen in children and young adults, especially in Asian countries.^8,9^ One type is characterized by clonal proliferation of EBV-infected cytotoxic T cells.^9^ A recent study by Lim et al looked at HLH patients with hemophagocytosis in their bone marrow. The authors found that 76% of EBV DNA positive patients and 85% of patient with positive EBV from in situ hybridization had malignancy-associated HLH, highlighting a significant correlation with EBV infection, malignancy, and HLH.^7^ In our patient malignancy was never ruled out given the family decline for lymph node biopsy. It can be speculated that if our patient was exposed to EBV in his home country of Laos, this may have led to the development of a lymphoproliferative disease and subsequently HLH, which could have contributed to his overall poor prognosis and outcome. However, we do not have enough data from the case to fully substantiate this hypothesis. An additional theory discusses the mechanisms of defective T cells and NK cells to properly remove antigen resulting in ongoing uncontrolled macrophage activity.^2,4^ These proposed mechanisms are part of the criteria used to diagnose HLH (Table 2).

Not only is diagnosis difficult and often elusive, but the differential diagnosis of HLH is broad. Three major groups lead the differential, including macrophage activation syndrome, septic shock, and immune system malignancies. Macrophage activation syndrome is a considered to be a type of HLH that is specifically caused by autoimmune disorders including systemic juvenile idiopathic arthritis in children and Still’s disease in adults.^4^ In the intensive care unit setting, HLH may appear exactly like septic shock and multiorgan dysfunction syndrome. As discussed above, a high index of suspicion is needed to differentiate HLH from septic shock, and often both HLH and septic shock are treated simultaneously. Some malignancies such as lymphoma and Langerhans cell histiocytosis can also present like HLH.^2^

Ferritin, although mostly known for its role in iron storage regulation, is elevated in many inflammatory states. Ferritin levels increase due to pro-inflammatory cytokine signaling; as such, ferritin has been a target of study to determine its significance in the diagnosis of HLH.^6^ The 2004 diagnostic HLH criteria includes ferritin levels greater than 500 ng/mL; however, as lower elevated ferritin levels have been associated with other inflammatory diseases, the cutoff level for HLH has been proposed to be higher than 500 ng/mL.^2,6,10,11^ A 2008 pediatric study by Allen et al showed that ferritin levels >10 000 µg/L had a 90% sensitivity and 96% specificity for HLH, and the addition of clinical signs such as fever increased the specificity to 98%.^6^ The same group later performed a study with a larger number of HLH patients and found that 93.2% of patient diagnosed with HLH in their study had ferritin levels >2000 µg/L.^11^ Aside from its diagnostic value, ferritin has also been studied for its prognostic value. In previous literature, it has been shown that higher ferritin at time of diagnosis as well as a slower rate of decrease in ferritin is associated with a poor prognosis and higher mortality.^1,2^ Although interleukin (IL)-2 level and natural killer (NK) cell activity are potentially more specific biomarkers of HLH, ferritin is more readily available and a high level can offer the first step to an earlier diagnosis of HLH.

A key pathologic finding of HLH is the presence of hemophagocytosis in bone marrow, lymph nodes, or the spleen; however, it is not pathognomonic with studies showing a sensitivity of 80% to 83% and a specificity of only 60%.^2^ Hemophagocytosis can be seen is several other diseases including viral infections, especially EBV.^7^ It can also be seen in various hematologic malignancies, with the most common being non-Hodgkin’s lymphoma.^7^ In the initial stages of HLH, hemophagocytosis may not be present and thus initial bone marrow biopsies may be falsely negative.^1,12^ The absence of hemophagocytosis does not exclude the diagnosis of HLH; however, its presence alone is not sufficient for diagnosis. In the correct clinical setting and among other laboratory findings, hemophagocytosis can greatly aid in diagnosing HLH.

Prior to the initiation of the HLH-1994 and later HLH-2004 treatment protocols, the mortality of untreated HLH was 95% with a median survival of less than 2 months, making early recognition and prompt treatment imperative.^2^ The original HLH-1994 treatment protocol recommended an 8-week induction with IV dexamethasone and etoposide, and the most recent HLH-2004 protocol added cyclosporine to help prevent relapse.^13^ In cases of primary HLH, relapsed HLH, or HLH from an unknown cause, hematopoietic stem cell transplant is ultimately recommended to improve long-term prognosis.^2,13^ Of note, etoposide is used to treat HLH due to its ability to target activated immune cells and decrease the characteristic IL-2-driven storm.^14^ Early initiation of etoposide, within 4 weeks of diagnosis, has been shown to improve long-term survival specifically in cases of EBV-HLH (90% in patient receiving early etoposide vs 57% in patient who did not receive treatment or delayed treatment) due to its ability to hinder lymphocytes activated by EBV.^2,4^ However, it is recognized that the database for using any agents to treat HLH is limited, and ultimately HLH treatment needs to be customized to each patient after appropriate specialist consultation.

Our patient did not receive etoposide due to his liver failure and clinical decline. However, he did receive rituximab based on a study in 2013 showing that the rituximab in conjunction with the HLH-2004 treatment protocol helps decrease inflammation and improve clinical symptoms.^5^ The proposed mechanism of rituximab in EBV-induced HLH is that it depletes B cells, ultimately removing the substrate for inappropriate EBV activation.^5^ Unfortunately, despite dexamethasone and rituximab treatment, our patient continued to decline and ultimately died.

The initial clinical presentation of septic shock and HLH can be similar with fever, nonspecific symptoms, and multiorgan failure. Additionally, a close look at the pathophysiology of sepsis and HLH reveal remarkable similarities. Infection results in immune activation of T lymphocytes and macrophages that release cytokines much like the immune-triggering mechanisms seen in HLH.^12^ Once the infectious source has been removed or treated, NK cell activity induces apoptosis in the activated lymphocytes and terminate the process.^12^ The difference between infection and HLH is that decreased NK cell activity allows the inflammatory response to continue uncontrolled.^12^ It has been proposed that perhaps HLH is not a stand-alone disease but part of the extreme end of the inflammatory sepsis spectrum.^12,15^ In older literature, several cases were looked at in which patients presented with fever and multiorgan failure and were presumptively diagnosed with septic shock. Despite broad-spectrum antibiotics, no obvious sources of infection were found, and the patients continued to decline without response.^12^ These patients progressively worsened and developed pancytopenia with eventual bone marrow biopsy leading to the diagnosis of HLH.^12^ Therefore in patients with a sepsis-like presentation, no obvious infectious source, and minimal response to standard treatment, one should search early for HLH. This should include further laboratory testing and possibly a bone marrow biopsy in keeping with the diagnostic criteria for HLH.

## Conclusion

Acquired HLH is a rare but frequently fatal disease seen in adults, often triggered by infection or hematologic malignancies. Diagnosis is challenging as clinical and laboratory findings are often nonspecific, and HLH can mimic the very diseases that trigger it. An extremely elevated ferritin level can be an early laboratory finding that raises suspicion for HLH, as was the case in our patient. Furthermore, in patients presenting with presumed severe sepsis/septic shock with no clear source, HLH should be considered on the differential. Early recognition and initiation of treatment is critical to help improve survival.
